# Disentangling effects of disturbance severity and frequency: Does bioindication really work?

**DOI:** 10.1002/ece3.7019

**Published:** 2020-12-19

**Authors:** Remigiusz Pielech, Patryk Czortek

**Affiliations:** ^1^ Department of Forest Biodiversity University of Agriculture in Krakow Kraków Poland; ^2^ Foundation for Biodiversity Research Wrocław Poland; ^3^ Faculty of Biology Białowieża Geobotanical Station University of Warsaw Białowieża Poland

**Keywords:** bioindication, community ecology, disturbance, ecological indicator, flood, riparian forests

## Abstract

Ecological disturbances are recognized as a crucial factor influencing the attributes of ecological communities. Depending on the specific adaptation or life cycle, plant species show different responses to disturbances of different magnitudes. Herben et al. (*Journal of Vegetation Science*, 27, 628–636) proposed six disturbance indicator values (DIVs) that describe the niches of Central‐European plant species along gradients of disturbance frequency and severity. Here, we ask if the DIVs can be used in community ecology for bioindication of disturbance regime?

We used a dataset of riparian forests sampled within mountain catchments (the Sudetes, SW Poland). As the regime of disturbance is driven by changes in floods from the spring toward the mouth, we calculated the position of every plot along longitudinal (upstream–downstream) gradient and used it as a proxy for the disturbance severity and frequency. We then calculated the community‐weighted means (CWMs) for each of the six indices for each plot and analyzed whether these indices reflected the position of the plots along the rivers. We expected an increase in the severity indices and a decrease in the frequency indices downstream along the rivers. Moreover, we analyzed relationships between disturbance indices and species optima along longitudinal gradient.

Surprisingly, means for all analyzed indices increased along the rivers. Severity indices showed the strongest association with the longitudinal gradient. The disturbance severity index for herbs was the only index that differed significantly among species with different responses along longitudinal gradient. On these results, we identified a strong correlation between the severity and frequency indices as the main problem.

We conclude that the DIVs have considerable applicative potential; however, the determination of ecological niches separately for disturbance severity and frequency is difficult because different components interact to shape the realized niche of each species. All analyzed indices encompass different attributes of the disturbance regime including both severity and frequency.

## Graphical abstract

We tested disturbance indicator values (DIVs) for its potential use in community ecology for bioindication of disturbance regime. The DIVs have considerable applicative potential; however, the determination of ecological niches separately for disturbance severity and frequency is difficult because different components interact to shape the realized niche of each species. All analyzed indices encompass different attributes of the disturbance regime including both severity and frequency.

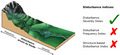

## 1 INTRODUCTION

Ecological disturbances have long been recognized as key components of ecological systems (Sousa, 1984; Turner, 2010). Conceptualized as “any relatively discrete event in time that disrupts ecosystem, community, or population structure and changes resources, substrate availability, or the physical environment” (Pickett & White, 1985), disturbance can seriously affect many ecological processes, for example, primary and secondary production, the accumulation of biomass, energy flow, and nutrient cycling. Different types of natural disturbances have been identified, including fires, flooding, windstorms, and insect outbreaks, as probably the most common worldwide. Regardless of their origin or agent type, a disturbance regime can be characterized by its frequency, spatial distribution, return interval, rotation period, disturbance size, intensity, and severity (Shea et al., 2004; Turner, 2010).

Although a vast body of studies has shown how different disturbance types affect vegetation across different spatial scales, the measurement and quantification of some characteristics of disturbances remain a challenge, especially in terms of disturbance intensity. For example, in riparian ecosystems, it is difficult to measure directly the amount of energy exerted by running water that influences the streamside vegetation during flood events. Instead, studies on the effects of disturbance intensity on riparian vegetation usually require either an experimental approach (Garssen et al., 2017; Konrad et al., 2011; Kotowski et al., 2010; Levine & Stromberg, 2001) or indirect methods, including spatial modeling of stream power (Bendix, 1999; Pielech et al., 2015), niche partitioning among hydrogeomorphic structures (Kyle & Leishman, 2009; Stoffel & Wilford, 2012), measurement of the amount of biomass removed by disturbance events (Eck et al., 2004; Garssen et al., 2015), or analysis of patterns of plant distribution along the longitudinal and lateral gradients (Araujo Calçada et al., 2015; Lite et al., 2005; Renöfält et al., 2005).

Recently, Herben et al. (2016) proposed a set of indicator values that describe the niche of each Central‐European plant species along the gradients of the disturbance frequency and severity. Disturbance indicator values (hereinafter referred to as DIVs) have been used as plant attributes in studies on plant life strategies (Bitomský et al., 2019; Herben et al., 2018; Klimešová et al., 2017). However, similar tools have been successfully applied to not only plant ecology but also in community ecology. For example, the Ellenberg's indicator values (EIVs, Ellenberg et al., 1992) describe the habitat preferences of Central‐European vascular plants by placing them on defined 9‐point (or 12‐point) scale for seven environmental variables, including soil moisture, pH, nutrients, light, temperature, continentality, and salinity. The EIVs are broadly used to calculate the mean values for whole communities and then characterize the habitat of these communities as a substitute for direct measurements (Diekmann, 2003; Pielech et al., 2017; Zelený & Schaffers, 2012). In this study, we ask whether DIVs can be used in a similar way to EIVs in community ecology for the bioindication of the disturbance regime? To test the applicability of DIVs, we used riparian forests as a study system. Riparian ecosystems occur along river banks and are defined as interfaces between aquatic and terrestrial habitats (Naiman & Décamps, 1997). They have been recognized as crucial ecosystems for maintaining regional biodiversity and providing essential ecosystem services (Dufour et al., 2018; Wierzcholska et al., 2018). They are also among the most threatened and degraded ecosystems, and numerous approaches have been adopted to assess the quality of riparian habitats. For example, Testi et al. (2012) examined the applicability of five different indicators, including the concept of indicator values of plants, in the assessment of the quality of riverine ecosystems.

Riparian forests grow along rivers and are therefore strongly influenced by flooding (Benda et al., 2004; Foster et al., 2020; Johnson et al., 2000; Naiman et al., 2005). Natural disturbances driven by floods shape species composition and richness as well as spatial structure in riparian ecosystems (James et al., 2016; Vervuren et al., 2003; You & Liu, 2018). However, flooding intensity, frequency, and duration differ in space and time. In riparian landscapes, water flows and flood disturbance vary along the river (Bazzaz, 1983; Gomi et al., 2002; Ito & Ito, 2008; Lite et al., 2005; Schlosser, 1990). Scaling up to watersheds, a cumulative effect due to the hierarchical structure of the watershed also shapes the spatial patterns of variation in the effects of floods. As a result, the patterns of flood disturbance vary mainly according to the distance from the river's source (Lepori & Hjerdt, 2006). Along the upper reaches (headwaters), the frequency of floods is the highest as most of the heavy rainfall results in a rapid increase in the amount of water that overflows the channel. Although frequent, local floods along the upper reaches affect only confined areas of the watershed and tend to be of low intensity to generate floods of modest duration and magnitude (Kochel, 1988; Lepori & Hjerdt, 2006). In addition, small local floods have only a small impact on the streamside vegetation. The overflow submerges plants for only a short period due to rapid water run‐off as a result of the slope of the valley bottom in the mountain valley. Contrary to this, the frequency of floods along the lower river reaches is lower because the climatic events that can affect entire watersheds are infrequent, and the asynchronous flooding of tributaries mitigates discharge variation downstream along a river (Lepori & Hjerdt, 2006). However, floods in the lower reaches tend to be of significant magnitude because the catchment areas are large. As the amount of water that overflows is large, floods in lower reaches cause severe damage to the floodplain's vegetation. In addition, these massive floods cause prolonged inundation that in turn eliminates plants that are susceptible to anoxia (Dittert et al., 2006; Renöfält et al., 2007). The mid‐reaches are thus subjected to floods of moderate severity and frequency, and the intermediate disturbance hypothesis (Connell, 1978; Grime, 1973; Shea et al., 2004) is usually used to explain the highest diversity in communities associated with the mid‐reaches (Lite et al., 2005; Nilsson et al., 1989; Renöfält et al., 2005; Tabacchi et al., 1996).

**FIGURE 1:**
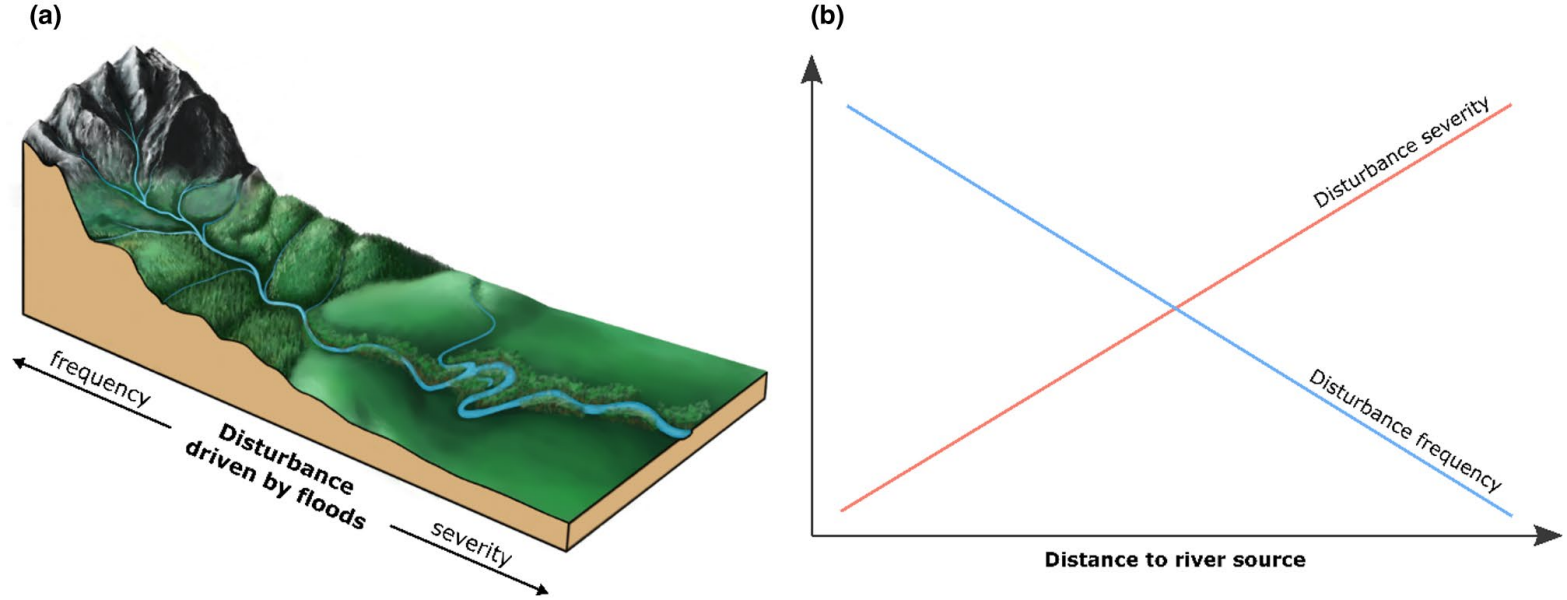
Conceptual model of spatial pattern of disturbance driven by floods along upstream–downstream gradient within studied area. Disturbance severity increases downstream along river, while disturbance frequency decreases in same direction. (a) Profile of river valley; (b) relationship between disturbance severity and frequency

The above‐mentioned relationships are also consistent with the flood pulse concept (FPC, Junk et al., 1989; Tockner et al., 2000). The FPC predicts that headwater streams are subjected to flooding regimes that are the result of local precipitation events, and therefore are characterized by low amplitude and high frequency. On the contrary, large‐river floods are driven by seasonal precipitation regime in the entire contributing area and therefore are characterized by high amplitude and low‐frequency variations in the water table (Couto et al., 2017). Thus, at the coarse spatial scale, the spatial variation in flood disturbances can be generalized as follows: Disturbance severity increases downstream along the river, while disturbance frequency increases upstream (Figure 1). This pattern is complicated at a finer spatial scale due to variations in geology and topography. However, the simplified conceptual model of spatial variation in disturbances driven by floods gives a unique opportunity to address ecological questions related to both disturbance frequency and severity. Here, we use a dataset of riparian forests sampled in mountain catchments in Southern Poland and calculate the community‐weighted means of the DIVs to determine whether these indicators reflect the spatial patterns of the disturbance frequency and severity along the rivers as described above. We expected an upstream increase in the community means of indices related to the disturbance frequency and downstream increase in the community means of indices related to disturbance severity.

**FIGURE 2:**
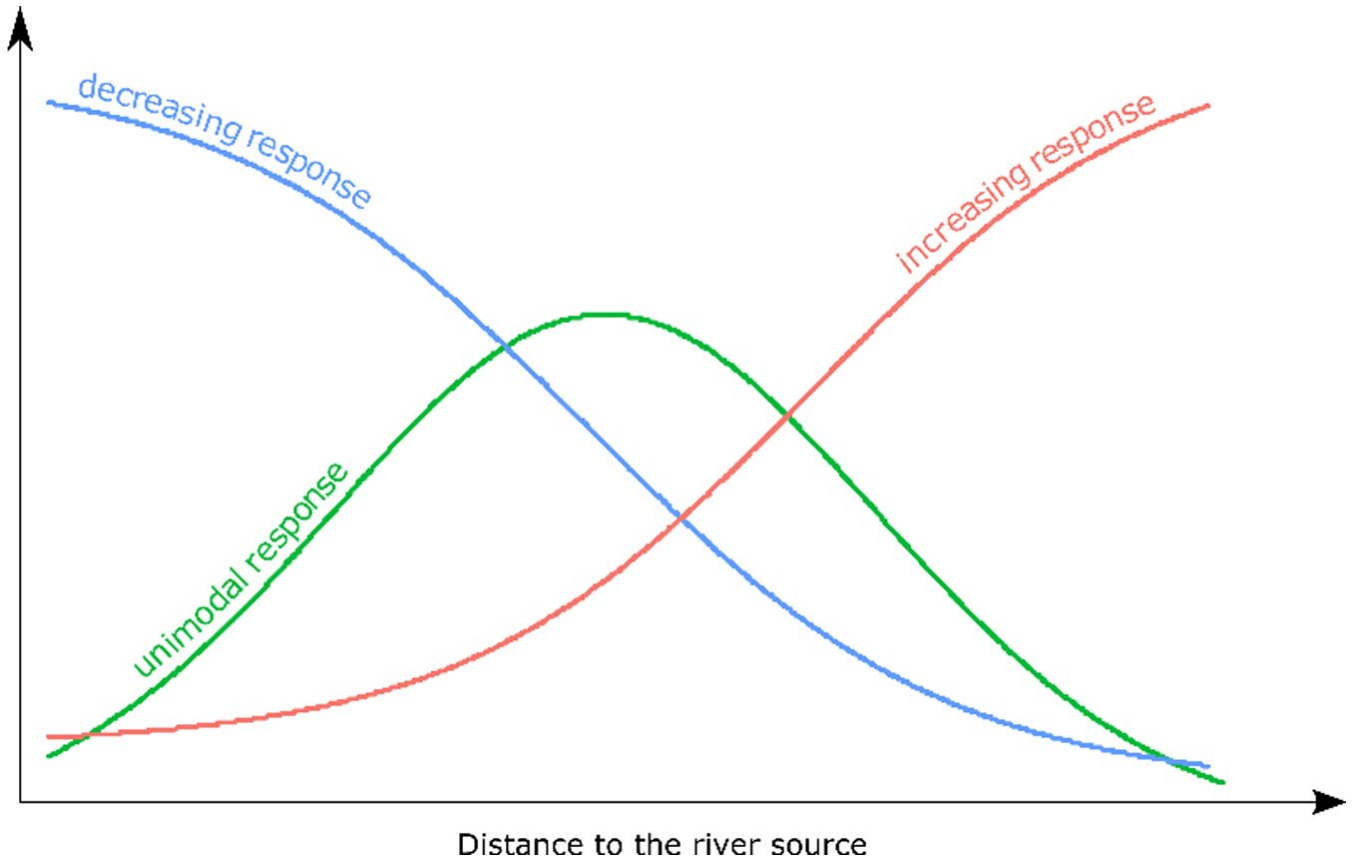
Three different response types of plants along longitudinal gradient identified on basis of previous study (Pielech et al., 2015)

In addition, we tested whether the DIVs reflect the ecological optima of the plant species along the longitudinal gradient (the term “longitudinal gradient” refers to the position along the river and is also referred to as the “upstream–downstream gradient”). For this, we compared differences in mean DIVs among three groups of plants, which revealed the different response types along the longitudinal gradient (Figure 2). Those groups were identified in a previous study on the riparian forests in the Sudetes (Pielech et al., 2015) based on Huisman–Olff–Fresco (HOF) modeling (Huisman et al., 1993). We expected that species having optimum along the headwater streams and decreasing downstream would have significantly lower values of the indices related to the disturbance severity compared with the plants with optimum along the mid‐reaches, which in turn have significantly lower values compared to the plants with optimum along the lower reaches. For indices related to the disturbance frequency, we expected the opposite lineup.

## 2 METHODS

### 2.1 Dataset and study area

The riparian forests were surveyed along mountain rivers in the Polish part of the Sudetes, SW Poland. The Sudetes cover an area of about 4,000 km^2^, and the elevation ranges from 300 (lower limit of the study area) to 1,602 m above sea level. The highest recognized sites of riparian forests in this mountain range are located at about 900 m a.s.l. The riparian forests of the studied area were subject to a detailed phytosociological survey, and seven different communities were distinguished (Pielech, 2015).

The dataset used in this study was collected between 2006 and 2009 following the methods of the Central‐European phytosociology (Dzwonko, 2007; Kent, 2012). All plots were located close to the river bank; plots were rectangular (7.5 × 20 m) and oriented with the long dimension parallel to the upstream–downstream gradient of the river. The cover of vascular plants was estimated for each of three layers (trees, shrubs, and herbs) using the Domin‐Krajina scale with an ordinal transformation proposed by van der Maarel (1979). Plots were also located with a GPS receiver and recorded in a mobile GIS database. The precise localization and digital river network (Map of Hydrographic Division of Poland, MPHP) enabled the calculation of the distance from the river source for all the sampled plots. More details regarding study area and vegetation sampling are available in the previous study (Pielech et al., 2015). The dataset is stored in the Forest Database of Southern Poland (Pielech et al., 2018).

### 2.2 Disturbance indices calculation

Herben et al. (2016) proposed a set of six indices that defined the niches of plant species along gradients generated by different components of the disturbance regime. These indices include two variants of disturbance frequency index, disturbance severity index, and structure‐based disturbance index. A disturbance frequency index (hereinafter referred to as DFI) indicates the species' adaptation to frequent disturbances, and a higher DFI indicates better adaptation. Similarly, a disturbance severity index (hereinafter referred to as DSI) indicates the species' adaptation to survive a severe disturbance. The DFIs and DSIs were calculated based on knowledge about the affinity of each species to the phytosociological classes. The authors assumed that all communities in each class are likely to be exposed to similar disturbance regimes. All vegetation classes were therefore characterized subjectively by estimating their disturbance severity and return time. To calculate the disturbance indices for each species, the authors used a large phytosociological database containing more than 30,000 vegetation plots and representing 39 phytosociological classes. Each class was characterized by an estimation of the disturbance frequency (mean number of years between two consecutive disturbances), disturbance severity (percentage of above‐ground biomass removed in a single disturbance event), and degree of soil disturbance (proportional change in cover of bare ground in a single disturbance event). A broad variety of disturbances was considered, including both natural and anthropogenic ones. For each species, the authors calculated the community‐weighted means of these three estimates (disturbance severity, disturbance frequency, and soil disturbance). The DFI was then calculated as the mean of the common logarithm of the disturbance frequency of all vegetation classes weighted by the occurrence frequencies of that species in those classes. A DSI was defined as the PCA‐derived shared variation in the mean disturbance severity and mean soil disturbance of all vegetation classes, weighted by the occurrence frequencies of this species in those classes. For forest vegetation, two variants of DFI and DSI were calculated including either the whole community or the herb layer only.

In addition, the structure‐based disturbance index (hereinafter referred to as SbDI) was calculated based on the structural characteristics of the vegetation. For each plot, the authors calculated the mean height at maturity of all the plant species recorded, *SD* of mean height at maturity, and the sum of the percentage covers of all species recorded within a plot. In the following step, the means, SDs, and variation coefficients were calculated for every species in a dataset. These calculations were then used in redundancy analysis (RDA); SbDI was defined as the combination of one height‐based variable and one summed cover‐based variable that yielded the best prediction of the first RDA axis. Like the DFI and DSI, the SbDI was calculated for two variants including either the whole community or herb layer only.

In this study, the dataset used all six disturbance indices proposed by Herben et al. (2016). For each riparian forest sample in our dataset, we calculated the community‐weighted means of these indices, that is, the means of indices of all species within a plot and weighted by the cover of those species. We expected an increase in the DSI and SbDI with the distance from the source and a decrease in the DFI with an increasing distance from the river source.

### 2.3 Statistical analyses

To explore the main patterns in the species composition of the vegetation plots along the longitudinal gradient, we used an ordination technique. The preliminary detrended correspondence analysis (DCA) showed a relatively long gradient length (>3 *SD*), and therefore, we decided to use detrended correspondence analysis as the most appropriate ordination method (Jongman et al., 1995; Lepš & Šmilauer, 2003). To perform the DCA, we used the *vegan::decorana()* function (Oksanen et al., 2017). To determine whether the DIVs reflect the patterns of disturbances along the longitudinal gradient, we calculated the community‐weighted means (CWMs) for all indices for each plot, and then, we passively fitted these CWMs as variables (vectors) into the ordination space using the *vegan::envfit()* function. For each variable, we calculated the determination coefficient *R* ^2^ and *p*‐value using a permutation test with 999 iterations. The significance of the results was evaluated at an alpha = 0.05. We performed two types of DCA analyses. The first included DSI, DFI, and SbDI for the herb layer only, and the second included the same set of indices for the whole community (including tree and herb layers).

We used linear regression to analyze the relationships between the position of the plot along the longitudinal gradient and the mean values of the DIVs (community‐weighted means). We analyzed each of the six disturbance indices with a maximum level of significance set at an alpha = 0.05. In addition, we also tested if the DIVs reflect the optima of the plant species along the longitudinal gradient. Here, we used the results of a previous study (Pielech et al., 2015) that recognized three types of response to the longitudinal gradient based on Huisman–Olff–Fresco (HOF) modeling (Huisman et al., 1993). Species with a “decreasing response” decreased their probability of occurrence downstream along the river, whereas species representing the "increasing response" increased their probability of occurrence downstream and were the most common in the lower reaches of rivers (Figure 2). The plants with the "unimodal response" were the most common in the middle reaches of the rivers (the middle of the longitudinal gradient analyzed). We used two approaches to analyze the links between the plant optima and the DIVs. First, we used linear regression to examine the links between the value of the species' optimum along the longitudinal gradient (which was calculated by the HOF model) and each of six DIVs. We expected that the species optima are positively correlated with DSIs and negatively correlated with the DFI. Second, we used an analysis of variance (ANOVA) to test whether there were significant differences in the DIVs between the groups of species with different types of responses. We also evaluated the significance of differences between the three groups of species analyzed using the post hoc Tukey test with a maximum level of significance set at an alpha = 0.05. All analyses were performed using R software (R Core Team, 2018).

## 3 RESULTS

**FIGURE 3:**
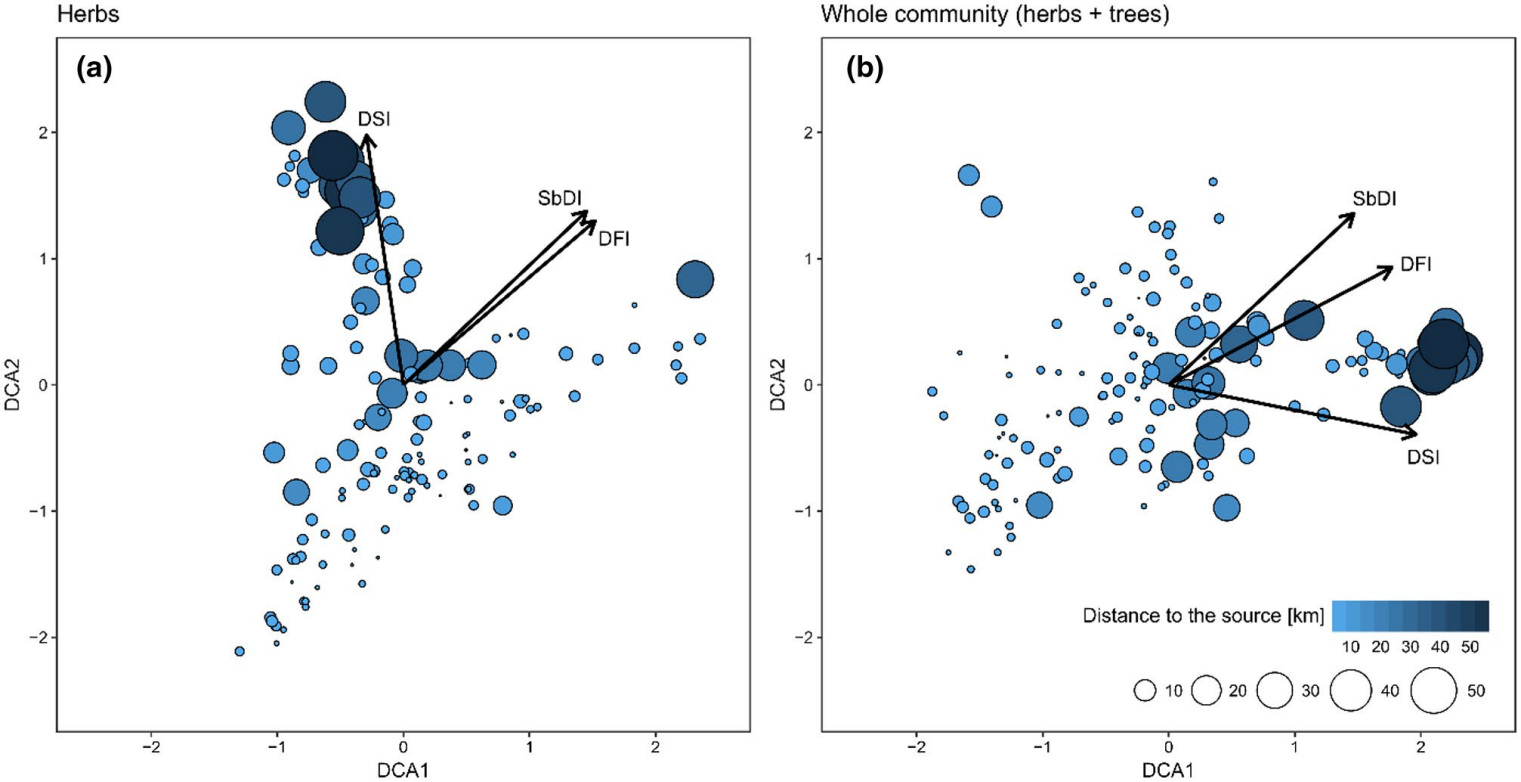
DCA biplots for herb layer (a) and whole community (herb and tree layers) (b) of riparian forests. Arrows represent gradients of passively fitted community‐weighted means of DIVs. Size and color of circle reflect distance to river source

The DCA for the herb layers revealed that the longitudinal gradient was associated with the second DCA axis (Figure 3a). Although the importance of the longitudinal gradient is evident, this result suggests that there is another essential gradient associated with the first axis. Analysis of the distribution of plots and species in ordinal space revealed that the first axis is associated with soil moisture. The DSI had the strongest link with the second DCA axis. The value of this indicator increased along the second DCA axis with the distance from the river source. The two remaining variables (DFI and SbDI) were strongly positively correlated with each other but showed only a weak correlation with the two first DCA axes.

The DCA for the whole community revealed that the longitudinal gradient was associated with the first axis. The gradient represented by the second axis could not be directly attributed to any known environmental factor or spatial gradient. Analysis of disturbance indicators revealed that the DSI had the strongest link with the first axis. The value of this indicator increased with the distance from the source. Two other indicators (DFI and SbDI) were strongly correlated with each other and showed the same direction, but the relationship with the first DCA axis was weaker (Figure 3b). The detailed parameters of the fitted variables for both DCA analyses are given in Table 1.

**Table 1:** Detailed parameters of passively fitted disturbance indices with DCA axes

Disturbance index	DCA1	DCA2	*p*	*R* ^2^
DFI for herb layer	0.7603	0.6495	.001	0.46
DSI for herb layer	−0.1465	0.9892	.001	0.93
SbDI for herb layer	0.7259	0.6878	.001	0.69
DFI for whole community	0.8849	0.4657	.001	0.79
DSI for whole community	0.9805	−0.1964	.001	0.83
SbDI for whole community	0.7341	0.6790	.001	0.76

**FIGURE 4:**
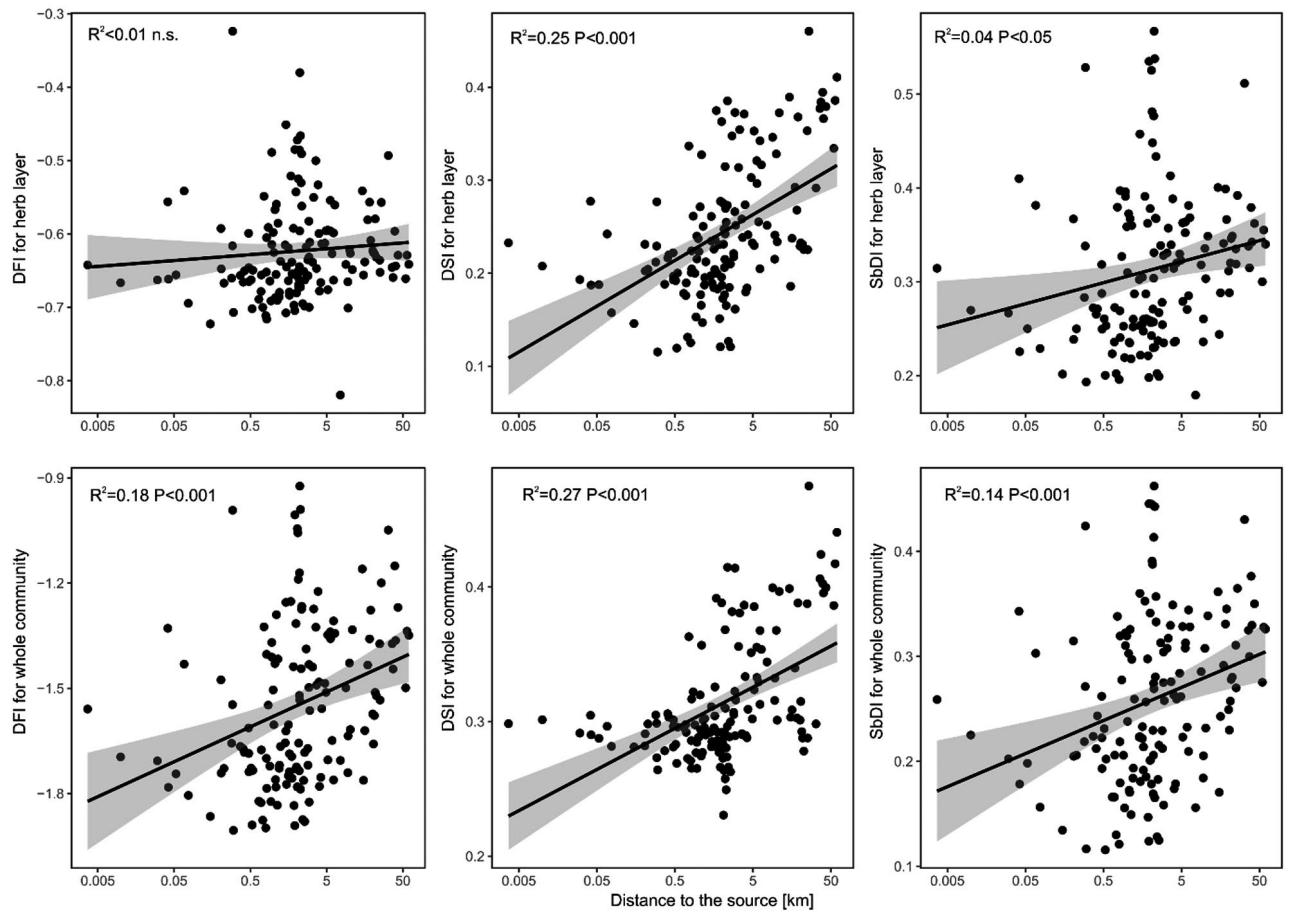
Relationships between localities of plots along longitudinal gradient and community‐weighted means of six disturbance indicators

We found that five out of the six analyzed disturbance indicators (except for the DFI for the whole community) significantly increased with increasing distance to the source. Both the DSI for herbs and for the whole community showed the strongest relationships with the distance to the source (*R* ^2^ = 0.25 and *R* ^2^ = 0.27, respectively). The SbDIs and DFIs showed weaker associations with the longitudinal gradient (Figure 4).

**FIGURE 5:**
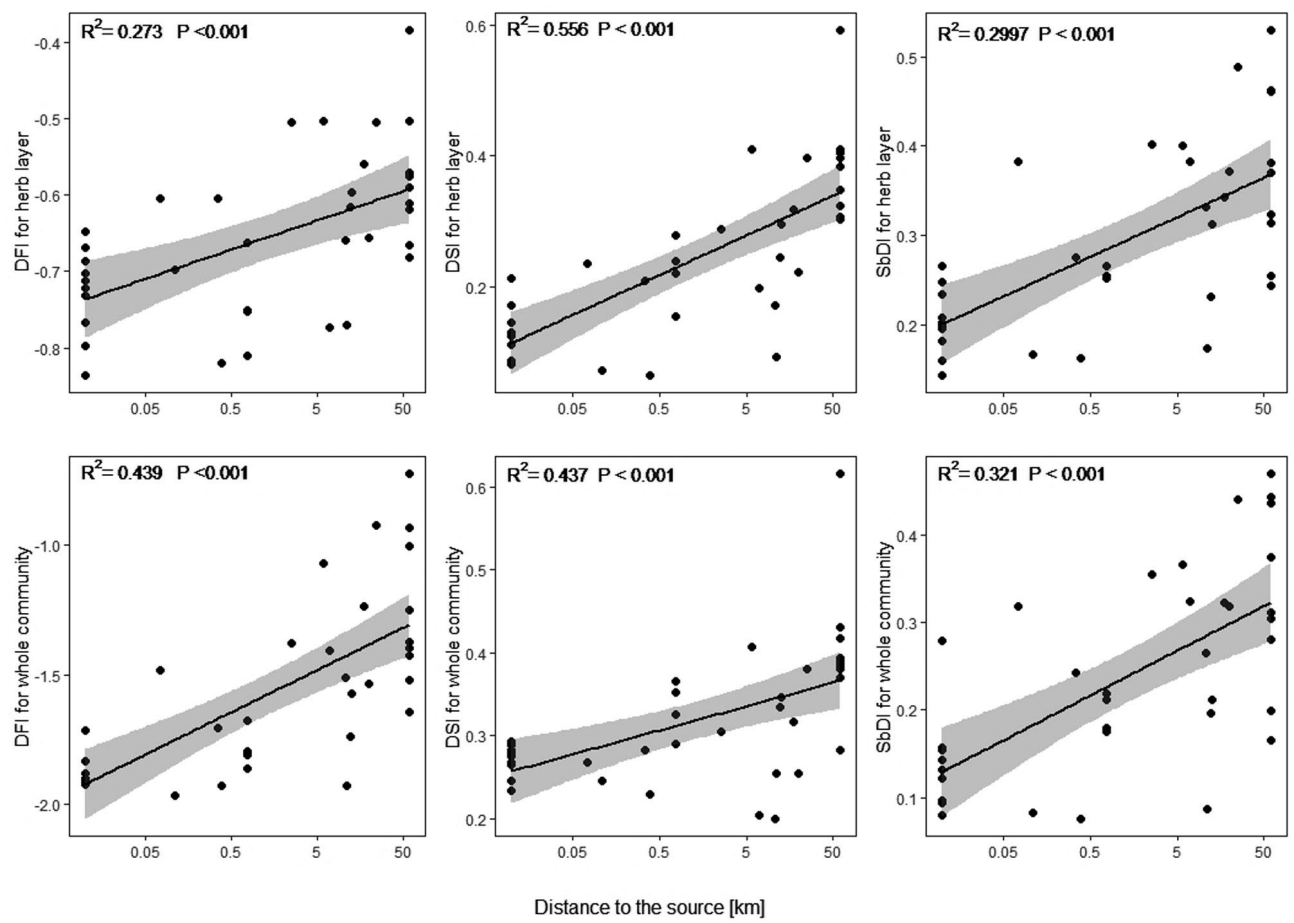
Relationships between species optima calculated by HOF modeling and values of six disturbance indicators

**FIGURE 6:**
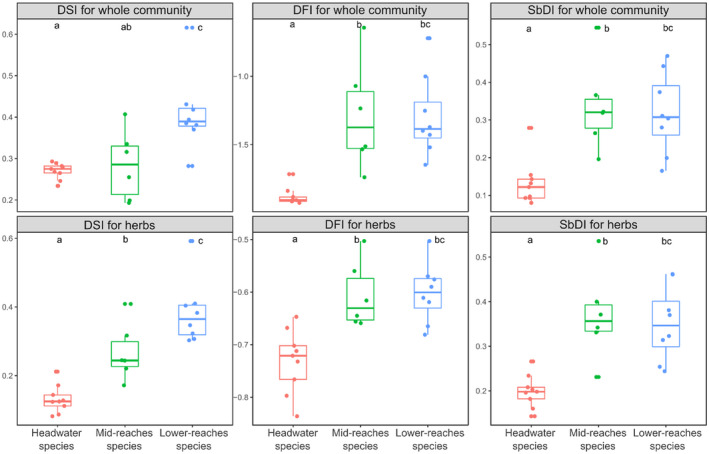
Comparison of values of six disturbance indices among groups of plants with different responses along longitudinal gradient (colors representing different response types are consistent with Figure 2)

As for the community‐weighted means, all six disturbance indices were positively correlated with the longitudinal gradient (Figure 5). In other words, the further from the river source a species' optimum, the higher the DIV. Contrary to our expectations, DFIs were also positively linked with the longitudinal gradient expressed by species optima. The comparison of the DIVs for species with different response types to the longitudinal gradient revealed that only the DSI for herbs reflected our initial expectation. Species with optima along the headwaters have significantly lower DSI values compared to species with optima along the mid‐reaches, which in turn have significantly lower DSI values compared to species with optima along the lower river reaches. All the other indices also showed similar patterns, but not all three groups differed significantly among each other (Figure 6).

## 4 DISCUSSION

Out of the three types of analyzed indices, only the disturbance severity index (DSI) revealed a tendency consistent with our initial expectation and expressed by the conceptual model presented in Figure 1. The community‐weighted means for the DSI were positively correlated with the distance from the river source and well expressed the general pattern of the disturbance severity along the river continuum within the studied area. Considering the two variants of DSI, the index calculated for the herb layer only performed much better in an indication of species responses to the longitudinal gradient. Species typical of small headwater streams, characterized by low‐severity floods, had the lowest values of the DSI, while the species characteristic of the lower reaches (the biggest rivers within the studied area) that were influenced by the most intensive and prolonged floods had the highest values of the DSI. It can be explained by the fact that a low‐intensity flood can influence the herbs and remove some of this biomass level; however, the trees are less prone to the low‐intensity flood‐driven disturbances and only a higher intensity of disturbance can cause serious damage. For the same reason, there is no difference in the tree diversity between the riparian forests along the headwaters and the mid‐reaches within the studied area. However, along the lower reaches the tree species adapted to the disturbances that prevail in many localities (Pielech, 2015). Finally, the DCA revealed that DSIs for the whole community and for herbs only were strongly correlated with the first and second axes in the ordinal space, respectively. This is consistent with the knowledge of the natural disturbance driven by floods as the main driver of species composition and structure in riparian landscapes (Biswas & Mallik, 2011; Stoffel & Wilford, 2012; Ward, 1998).

The DFI was not consistent with our conceptual model. First, the CWMs for this index increased with the distance from the source, thereby suggesting that disturbance frequency increased downstream along the river. Second, neither the DFI for the whole community nor for the herbs only were successful in distinguishing the species with a unimodal response and increasing response along the longitudinal gradient. These results led us to a detailed consideration of the relationships among the different components of the disturbance regime. We focused on the disturbance frequency and severity (or intensity as a feature strongly correlated with the disturbance severity). The indicators of disturbance frequency and severity calculated by Herben et al. (2016) were strongly positively correlated with each other (correlation coefficients for DSI versus. DFI for herbs and for the whole communities were 0.766 and 0.580, respectively). These relationships between the analyzed indices were expressed in our DCA; the DSI and DFI revealed a similar direction in the ordination space (Figure 3), thereby suggesting that—according to the DIVs—the disturbance frequency increased with increasing severity. In our study system, however, the direction of the frequency and severity was opposite; there were frequent disturbances of low severity along the upper river reaches and infrequent disturbances of high severity along the lower reaches. However, the opposite spatial pattern was also recognized, where the highest levels of disturbance severity and intensity were associated with the headwaters (e.g., due to the severe landslides along the high‐gradient headwater streams) and decreased downstream, while the disturbance frequency increased downstream (Ito & Ito, 2008; Malanson, 1993; Resh et al., 1988). Obviously, the generalizations about the longitudinal patterns of disturbance in the river networks are valid at a coarse landscape scale. Scaling down to local conditions, these general patterns may be distorted along the river at locations where the stream power peaks due to erratic changes in the slope and discharge or at the confluences (Benda et al., 2004; Lite et al., 2005). Regardless of the catchment characteristics, biogeographical settings, and spatial scales, there is one common factor for all the above‐mentioned cases: Severe disturbances occur less frequently than milder ones. This rule is also valid for other natural disturbances driven by different agents (Bazzaz, 1983; Turner et al., 1998). In addition, it has been shown that many types of natural disturbances exhibit an inverse relationship between intensity and frequency, whereas anthropogenic disturbances tend toward both high intensities and frequencies (Turner et al., 1989). In that light, a strong positive correlation of indices reflecting disturbance severity (DSI) and disturbance frequency (DFI) might be the reason for the unsatisfactory performance of the DFI.

Another problem that we identified is the question of how to quantify the disturbance frequency. For the purpose of the calculation of the DIVs, Herben et al. (2016) proposed to subjectively estimate the mean number of years between two consecutive disturbances common in each vegetation type. Although very intuitive, in our opinion, this approach may lead to a flawed estimation of species niches. First, disturbance frequency should be related to the recovery processes (regeneration) of the environmental system (Battisti et al., 2016). If the time needed for regeneration of the community after disturbance is much shorter than the period between two consecutive disturbances, this community may be inhabited by species prone to disturbance. Contrary to this, if the time required to complete the regeneration after the disturbance is shorter than the disturbance return interval, selective pressure exists, and species not adapted to this pressure are eliminated. Second, the disturbance frequency should be related to the life span and life cycle of the species that predominate in a given habitat (Bazzaz, 1983; Resh et al., 1988).

The above‐mentioned problems related to the disturbance frequency led us finally to the following question: Is it possible at all to define the niches of plant species separately for disturbance severity and disturbance frequency? It seems that the disturbance regime shapes the species' realized niche by an interaction of both the disturbance severity and frequency. The ecological disturbances influence the different attributes of a population, including diversity and dispersion, growth rate, and age structure. Moreover, disturbances are also responsible for selecting specific plant adaptations, biomass removal, limiting the soil seed bank, and changes in resource distributions. It is extremely difficult to assess which component (severity or frequency) of the disturbance regime controls these processes and attributes. Thus, some authors claim that the disturbance frequency should not be considered separately, but rather in the context of the disturbance intensity (Turner et al., 1998). Although the DSIs and DFIs tried to separate between the frequency and severity, we find that, to some extent, they encompass both and reflect the niche of each species along the complex gradient of the disturbance magnitude. From among the three different indices analyzed, the DSI performed the best in this matter and seemed to be a promising tool in community ecology. However, further tests of its applicability are needed on other vegetation types and with different disturbance agents considered.

In this study, we used a position along a river as a proxy for the gradient generated by the disturbance regime. However, one has to be aware that the longitudinal gradient is very complex and is related not just to the disturbance regime. Along the rivers, there is a continuous change in the various physical features of the environment, including water discharge, size of the channel, chemistry, availability of light, and organic matter and accumulation to erosion ratio, among others. All these factors interact, shaping the realized niche of the plant species along the rivers.

Finally, the DIVs were calculated on the basis of the broadly defined vegetation classes and some of them were very heterogeneous in terms of the disturbance regime. For example, the *Carpino‐Fagetea* class encompassed beech forests and oak‐hornbeam forests, which are disturbed infrequently, as well as forests of ravines and alluvial floodplains, which are characterized by frequent disturbances of diverse magnitudes. On the one hand, defining more homogeneous vegetation units could be beneficial for better delimitation of the realized niches of plants. While, on the other hand, defining more homogeneous groups could decrease the independence of the DIVs from the plant traits.

## 5 CONCLUSIONS

Disturbance indicator values (DIVs) have considerable applicative potential in community ecology. However, the determination of the ecological niche of plant species separately for disturbance severity and frequency seems to be very difficult because different components of the disturbance regime interact to delimit the realized niche of each species. Thus, we find that each of the three types of disturbance indices proposed by Herben et al. (2016) encompassed different attributes of the disturbance regime including both severity and frequency. In our study, which was performed at the landscape scale, the spatial pattern of the disturbance regime in the riparian forests was best reflected by the disturbance severity index (DSI). However, further tests of the applicability of DIVs are needed on other vegetation types and with different disturbance agents considered.

## CONFLICT OF INTEREST

The authors declare no competing interests.

## AUTHOR CONTRIBUTIONS

**Remigiusz Pielech:** Conceptualization (lead); data curation (equal); formal analysis (equal); funding acquisition (lead); investigation (lead); methodology (equal); project administration (equal); resources (lead); software (equal); supervision (lead); validation (equal); visualization (equal); writing—original draft (equal); writing—review and editing (equal). **Patryk Czortek:** Conceptualization (supporting); data curation (equal); formal analysis (equal); funding acquisition (supporting); investigation (supporting); methodology (equal); project administration (equal); resources (supporting); software (equal); supervision (supporting); validation (equal); visualization (equal); writing—original draft (equal); writing–review and editing (equal).

## Data Availability

The dataset analyzed in this study is accessible on the Dryad repository, https://doi.org/10.5061/dryad.bg79cnp8v. In addition, the extended dataset, including additional environmental variables and more sample plots, is stored in and available (on request) from the Forest Database of Southern Poland (Pielech et al., 2018).
